# Results of ultrasound-guided release of tarsal tunnel syndrome: a review of 81 cases with a minimum follow-up of 18 months

**DOI:** 10.1186/s13018-020-1559-1

**Published:** 2020-01-28

**Authors:** A. Iborra, M. Villanueva, P. Sanz-Ruiz

**Affiliations:** 1 Department of Podiatry, Faculty of Health Sciences, University of La Salle, Institute Avanfi, 28020 Madrid, Spain; 2 Avanfi Institute and Unit for Ultrasound-guided Surgery, Hospital Beata María Ana, Calle Orense 32, 1, 28020 Madrid, Spain; 3 Institute Avanfi, 28020 Madrid, Spain

**Keywords:** Tarsal tunnel syndrome, Heel pain syndrome, Ultrasound-guided surgery, Tibial nerve, Medial plantar nerve, Lateral plantar nerve

## Abstract

**Background:**

This study aims to analyse the clinical results of ultrasound-guided surgery for the decompression of the tibial nerve, including its distal medial and lateral branches, to treat tarsal tunnel syndrome. These structures are the complete flexor retinaculum and the deep fascia of the abductor hallucis muscle, including individualised release of the medial and lateral plantar nerve tunnels.

**Method:**

This is a retrospective review of 81 patients (36 men and 45 women) with an average age of 41 years old (32–62) and an average clinical course of 31 months (8–96) compatible with idiopathic tarsal tunnel syndrome, who underwent ultrasound-guided decompression of the proximal and distal tarsal tunnel between February 2015 and November 2017 (both months included), with a minimum follow-up of 18 months.

**Results:**

Based on the Takakura et al. scale for the 81 patients, 76.54% obtained excellent results, 13.58% good results, and 9.87% poor results.

The patients with the longest course of symptoms displayed the worst results.

**Conclusion:**

Although 9% of patients did not improve, ultrasound-guided tarsal tunnel release might be a viable alternative to conventional open approaches.

## Introduction

Fifteen percent of adults experience pain in the plantar region and heel at some point in their lifetimes. One of the causes of this pain is tarsal tunnel syndrome. The prevalence of tarsal tunnel syndrome (TTS) described in the literature is lower than that of other compressive neuropathies. However, some authors consider that it is underdiagnosed or occasionally misdiagnosed as plantar fasciitis [1].

TTS is a peripheral neuropathy caused by entrapment of the tibial nerve and its branches in the flexor retinaculum of the ankle located in the deep fascia of the abductor in the rearfoot [2, 3]. The first anatomical description of the tarsal tunnel is attributed to Richter in 1897, and the first clinical description of TTS was provided by Von Malisé [4] in 1918. Subsequently, in 1987, Heimkes et al. [5] described a distal tarsal tunnel syndrome, defined as compression from the deep portion of the abductor hallucis muscle (AHM) and its fascia, the confluence of the deep abductor fascia, and the medial edge of the plantar fascia, over the fascia, and by the quadratus plantae muscle. Meanwhile, the descriptive anatomy of this region was exhaustively described by Kelikian and Sarrafian [6]. Dellon and Mackinnon describe a high neuroanatomical variability of the tibial nerve and its branches, which could be related to the diagnostic difficulty of TTS [7].

The clinical course of TTS starts with pain in the heel and feet, a burning sensation, paraesthesia in the toes, heaviness in the sole of the foot, shoe pressure, and nocturnal symptoms [8]. Lengthy walks and standing still often exacerbate the symptoms [9]. One of the main problems in treating TTS is that it is difficult to diagnose. Supplementary examinations and tests used to diagnose nerve compression lack sensitivity. Therefore, there is an ongoing debate regarding the prevalence of the condition, the best time for surgical intervention, the surgical approach, and relapse management [9, 10]. Currently, three surgical procedures are indicated for decompression of the tibial nerve and its branches: open surgery, endoscopic surgery, and ultrasound-guided surgery [10, 11].

The authors have previously described the very high reliability of using high-resolution sonography to delineate the normal anatomy of the tibial nerve and its branches in the tarsal tunnel (even in branches measuring 1 mm in diameter) [12], and also in the surgical technique for decompression of the proximal and distal tarsal tunnel [11].

In this document, the authors present the results of ultrasound-guided decompression of the proximal and distal tarsal tunnel in patients with idiopathic TTS.

## Methods

We carried out a retrospective review of 81 patients who underwent ultrasound-guided surgery for TTS from 2015 to 2017. The minimum postoperative follow-up was 18 months.

The review included patients diagnosed with idiopathic TTS for whom conservative treatment had failed and who had not received simultaneous surgery for another pathology (e.g. gastrocnemius lengthening and plantar fasciitis). The review excluded patients with an intrinsic or extrinsic factor that could have caused tarsal tunnel compression, such as metabolic and autoimmune diseases, space-occupying lesions, and possible more proximal compressions (e.g. lumbar spine injuries), and patients undergoing combined surgical techniques, such as ultrasound-guided gastrocnemius lengthening, plantar fasciotomy, tibial nerve decompression in the soleal sling, subtalar implant, and calcaneal osteotomy to correct foot pronation.

The average age of the patients was 41 years old (32–62). There were 36 men and 45 women. Of the 81 patients, 16 were bilateral; hence, 97 ultrasound-guided proximal and distal tarsal tunnel decompressions were performed. A total of 93.8% (76) patients attended our clinic with a prior diagnosis of plantar fasciitis and an average clinical course of 31 months, having previously received conservative treatment, such as physiotherapy, custom-made plantar orthoses, shock wave therapy, steroid injections, and platelet-rich plasma, with unsatisfactory results. The remaining patients [5] presented with a primary diagnosis of tarsal tunnel syndrome.

All the patients displayed the classic symptoms of nerve compression (i.e. tingling, burning, irritation, and heaviness in the sole of the foot) and were positive for Hoffman-Tinel and Valleix signs. The EMG studies were considered inconclusive due to the high probability of false negatives [13]. Only patients with bilateral symptoms underwent or were prescribed an EMG in a battery of tests to rule out spinal or compressive diseases that could cause neuropathic pain in the feet. MRI and ultrasound scans were requested to rule out other pathologies and space-occupying lesions that could have compromised the nerve. Therefore, all the cases were diagnosed as idiopathic TTS [14].

### Surgical technique

Ultrasound-guided proximal and distal tarsal tunnel release was performed on all patients, following the technique previously described by the authors [15].

**Fig. 1:**
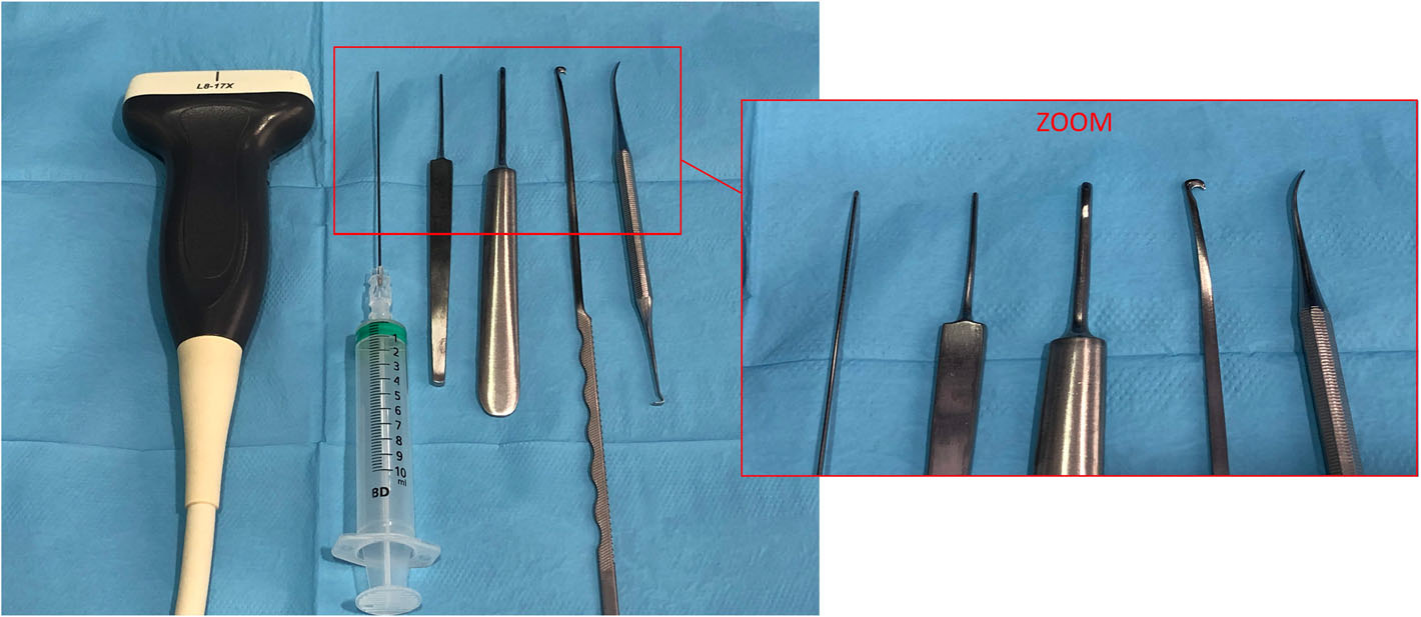
Set of instruments

The set of instruments included long needles (a 20-G, 0.9 × 90-mm-diameter BD spinal needle; Becton Dickinson S.A. Madrid, Spain), two V-shaped straight curettes no. 1 and no. 2, a blunt dissector, a 3-mm retrograde hook knife (Smith and Nephew), and an ultrasound device (Alpinion ECube15) with a 8–17-MHz linear transducer with the Needle Vision Plus™ software package (Alpinion Medical Systems, Bothell, WA, USA) (Fig. 1).

**Fig. 2:**
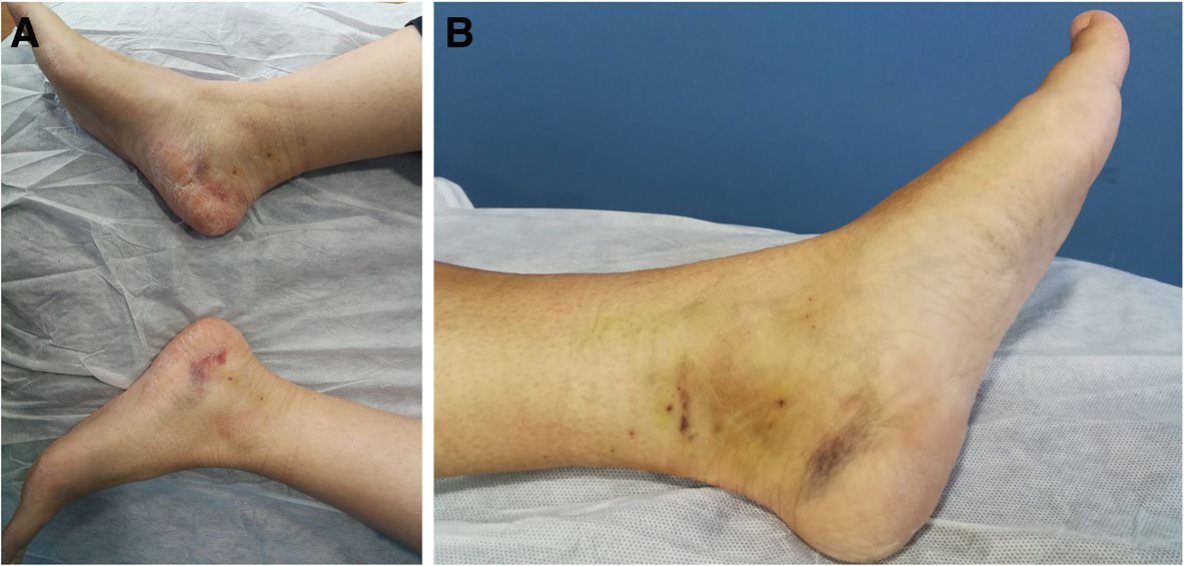
Bilateral proximal and distal tarsal tunnel decompression (**a**). Unilateral proximal and distal tarsal tunnel decompression (**b**)

The ultrasound-guided release of the proximal and distal tarsal tunnel was performed with two incisions (each measuring 1 to 2 mm) to release the flexor retinaculum and the deep fascia of the abductor hallucis muscle, thereby decompressing the four medial tunnels in the ankle (Fig. 2). The technique, which was performed on an outpatient basis, required local anaesthesia but no tourniquet. Following the operation, the patients left the hospital walking on crutches. Starting on the day of the surgery, the patients performed flexural and extension movements of the ankle to prevent perineural fibrosis and subsequent nerve entrapment. The rehabilitation programme commenced 4 days after surgery.

The outcome of the procedure was assessed using the Takakura et al. [3] scale: a simple grading system for comparing results before and after surgery. Scores were recorded using a 10-point scale to measure spontaneous pain, pain on movement, burning pain, Tinel’s sign, sensory disturbance, and muscular atrophy or weakness. Two points were assigned to the absence of each sign or symptom (Table 1). A total score of 10 points was considered excellent, 8 to 9 as good, 6 to 7 as fair, and under 5 as poor (Table 1). The score was calculated before surgery and at 3, 6, 12, and 18 months after the surgery.

**Table 1:** Rating scale for severity of tarsal tunnel syndrome and surgery from Takakura 1991; a normal foot scores 10 points

Symptoms	Absent	Some	Definite
Pain, spontaneous or on movement	2	1	0
Burning pain	2	1	0
Tinel’s sign	2	1	0
Sensory disturbance	2	1	0
Muscle atrophy or weakness	2	1	0

## Results

All the patients suffered from the classic symptoms of nerve compression, such as tingling, burning, irritation or heaviness in the sole of the foot, and positive Hoffman-Tinel and Valleix signs.

**Fig. 3:**
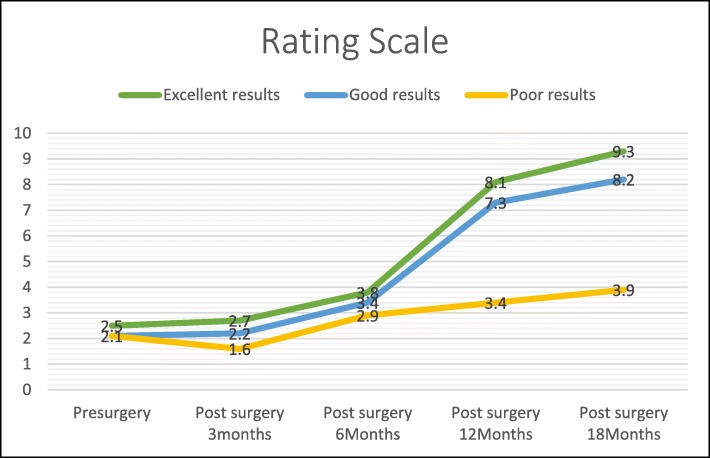
Results graph

A total of 76.54% (62) patients obtained excellent results, 13.58% (11) good results, and 9.87% (8) poor results on the Takakura scale (Fig. 3).

The course for both the excellent and good results over the 18 months shows that the score remained unchanged with no significant improvement in the first 6 months, after which the values improved from months 6 to 12 and continued to do so up to the 18-month follow-up (Fig. 3).

## Discussion

Fifteen percent of adults experience pain in the plantar region and heel at some point in their lifetimes. The causes include tarsal tunnel syndrome, which may be an underdiagnosed condition [1, 16]. Doneddu et al. [17] refer to a literature review that found that TTS was the fifth most commonly published nerve compression syndrome in the scientific literature from 1 January 2016 to 1 June 2016, with 134 articles, compared to 2450 indexed articles for carpal tunnel syndrome.

There are three methods for decompression of the tibial nerve and its branches: open surgery, endoscopic surgery, and ultrasound-guided surgery [11, 18].

According to the authors, the success rate of tarsal tunnel surgery with open or endoscopic decompression ranges from 44% to 96% [17, 19]. The variation in the results is primarily due to patient selection, clinical course duration, and surgical technique. Better results were observed in patients with space-occupying lesions. Some authors conclude that the surgical results are worse in idiopathic TTS and also when the clinical course exceeds 1 year [10]. By contrast, the results are more favourable when the course is less than 10 months, and the surgical technique aims to decompress the proximal and distal tarsal tunnel [20], thus decompressing three or four medial tarsal tunnels. It is important to explain to the patient that the symptoms of tingling, pain, and swelling may increase following surgery and can take up to a year to resolve completely while the nerve fibres regenerate and axon levels return to normal [21].

The documented postoperative complications of these techniques include impaired wound healing, infection, and keloid formation. Complex regional pain syndrome (CRPS) has also been reported as a rare sequela of surgery; however, lesions of the calcaneal branches can produce causalgia in the heel area [22].

To the best of our knowledge, this is the largest reported surgical series for tarsal tunnel syndrome and the first to describe the results of the ultrasound-guided release of the proximal and distal tarsal tunnels in TTS.

**Fig. 4:**
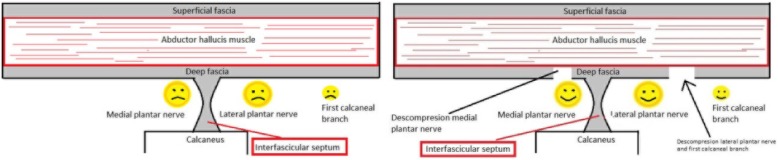
Point of the release and position of the main structures: compression of the MPN, LPN, and first calcaneal branch versus decompression of the MPN

Of the group of 81 patients who underwent ultrasound-guided tarsal tunnel release, the minimum postoperative follow-up was 18 months (3, 6, 12, and 18 months). In total, 76.54% of patients obtained excellent results, 13.58% good results, and 9.87% poor results, according to the Takakura scale (Fig. 3). These percentages are similar to those obtained by other authors who performed open or endoscopic decompression of the three or four tarsal tunnels [19, 23]. If we compare our results to those of Mullick et al. [19], with approximately the same sample size, our percentage of patients with excellent results (76.54%) is very similar to that obtained by Mullick at al. (82%). The authors attribute the good results to decompression of the four tarsal tunnels and performing opening and resection of the abductor hallucis septum to create one long distal tunnel. Our ultrasound-guided technique does not involve excision of the septum; instead, the deep fascia of the abductor is opened in the two distal tarsal tunnels and, thereby, decompressed and enlarged (Fig. 4).

This might represent a limitation in our ultrasound-guided surgical technique; however, the results are comparable to those obtained by other authors. In our practice, we reserve septum excision, associated with internal neurolysis of the nerves, for patients with poor results following ultrasound-guided release [24].

Mullick et al. [19] obtained a more favourable result in patients with symptoms lasting less than 10 months. Furthermore, Sammarco states that his results were time-dependent; in other words, the most satisfactory results were yielded from patients who have experienced symptoms for less than 1 year [20].

In our series, the mean course of the symptoms in the 81 patients was 31 months. The mean course was 22.6 months for patients with excellent results, 45.8 months for patients with good results, and 79.2 months for patients with poor results. In our clinical series, the patients with good and poor results had a longer clinical course, which is an important fact to bear in mind in the postoperative results of tarsal tunnel decompression. Therefore, as observed in studies conducted by other authors, our results are influenced by the duration of the clinical course; namely, the results were excellent in shorter courses and poor in longer courses. The eight patients with poor results required palliative treatment, such as radiofrequency, nerve block with bupivacaine, and steroids; five patients required open surgery to perform neurolysis and septum excision; and a further three patients underwent a neurotomy with a view to improving their quality of life [19].

The results obtained in patients with bilateral TTS symptoms were not encouraging, as only 2 of the 16 patients achieved excellent scores, 6 patients good scores, and 8 poor scores. Therefore, the review demonstrates that the outcome is worse for patients with bilateral symptoms as opposed to unilateral pathology. The poor results obtained by patients with bilateral pathology could be due to the fact that, although the tests for detecting rheumatic pathology were negative, they may have suffered from some hidden rheumatic disease affecting the peripheral nervous system.

One interesting finding is that Tinel’s sign intensified in all patients in the first month before later normalising to the preoperative intensity. This exacerbation is a positive postoperative indicator for a good clinical course, according to Ahmad et al. [10].

Another relevant finding is that the patients who obtained excellent or good results started to notice improvements from the sixth month (Fig. 3). Therefore, patients should be informed to expect a gradual recovery with a lessening of symptoms from 6 to 12 months after tarsal tunnel decompression surgery, rather than an immediate improvement. It has been suggested that the nerve fibres can take up to a year to regenerate and recover axon levels after decompression [17].

The most common postoperative complication was superficial haematoma formation, which was reduced intraoperatively by injecting adrenaline into the decompression pathways following release. The haematomas were spontaneously reabsorbed. In six feet, postoperative local anaesthesia occurred in the heel region, corresponding to the medial calcaneal branch, which resolved spontaneously with oral vitamin B (Hidroxil, B1-B6-B2, Almirall, Barcelona, Spain) for 2–3 months.

In the cases that produced poor results, an MRI scan was performed to assess whether there was postoperative fibrosis. Unlike open surgery, ultrasound-guided surgery did not show any signs of significant postoperative fibrosis, which can produce poor results [24, 25]. Similarly, our review did not reveal any problems related to dehiscence, poor healing, or complex regional pain syndrome, which is a major advantage of using the ultrasound-guided surgical technique for patients with venous insufficiency, diabetes, and other diseases that can delay healing.

## Conclusions

Ultrasound-guided proximal and distal tarsal tunnel release for TTS provides highly satisfactory results that are similar to conventional and endoscopic techniques with potentially fewer risks than these procedures as it does not require exsanguination of the leg and can be performed with local anaesthesia on an outpatient basis. A potential theoretical advantage is that it may reduce the risk of infection, wound complications, or fibrosis.

Another advantage of ultrasound-guided surgery over open or endoscopic techniques is that it can be performed bilaterally and in combination with other ultrasound-guided techniques, such as gastrocnemius lengthening and partial plantar fasciotomy [15, 26].

This innovative technique could be the first surgical option for TTS decompression, offering satisfactory results.

However, the learning curve is a long one, and the technique requires a high-resolution ultrasound scanner and training to ensure reliable ultrasound imaging. Therefore, further studies are required to determine the best time for surgery and whether a surgical variant, such as hallucis abductor septum section, might improve the results.

## Data Availability

The materials described in the manuscript, including all relevant raw data, are available from the first author upon request by email.

## Abbreviations

CRPS: Complex regional pain syndrome
EMG: Electromyogram
MRI: Magnetic resonance image
TTS: Tarsal tunnel syndrome
